# Defective support network: a major obstacle to coping for patients with heart failure: a qualitative study

**DOI:** 10.3402/gha.v9.30767

**Published:** 2016-04-01

**Authors:** Parvin Mangolian Shahrbabaki, Esmat Nouhi, Majid Kazemi, Fazlollah Ahmadi

**Affiliations:** 1 Razi School of Nursing and Midwifery, Kerman University of Medical Sciences, Kerman, Iran; 2 Physiology Research Center, Department of Medical Surgical Nursing, Razi School of Nursing and Midwifery, Kerman University of Medical Sciences, Kerman, Iran; 3 School of Nursing and Midwifery, Rafsanjan University of Medical Sciences, Rafsanjan, Iran; 4 Nursing Department, Faculty of Medical Sciences, Tarbiat Modares University, Tehran, Iran

**Keywords:** heart failure, coping, support network, obstacles, Lundman and Graneheim content analysis

## Abstract

**Background:**

Heart failure as a chronic disease poses many challenges for a patient in his or her everyday life. Support in various aspects of life positively affects coping strategies and influences the well-being and health outcomes of heart failure patients. Inadequate support may lead to a worsening of symptoms, increased hospital readmissions, psychological disorders, and a reduced quality of life.

**Objective:**

This study explored obstacles to coping related to support for heart failure patients as viewed by the patients themselves and their family members and caregivers.

**Design:**

This qualitative study was conducted using content analysis. The 20 Iranian participants included 11 patients with heart failure, three cardiologists, three nurses, and three family members of heart failure patients selected through purposive sampling. Data were collected through semi-structured interviews and analyzed using the Lundman and Graneheim qualitative content analysis method.

**Results:**

During data analysis, ‘defective support network’ developed as the main theme along with four other categories of ‘inadequate family performance’, ‘inadequate support by the healthcare team’, ‘distorted societal social support’, and ‘inadequate welfare support’.

**Conclusion:**

The findings of the current study can assist health authorities and planners in identifying the needs of patients with heart failure so as to focus and plan on facilitating their coping as much as possible by obviating the existing obstacles.

## Introduction

Heart failure is a chronic disease caused by cardiac dysfunction (1). It is a growing health problem in many countries as a result of the aging population (2). The number of heart failure patients in Iran is around 3,337 cases per 100,000 of the population (3). Most heart failure patients are the elderly, widows, and those socially isolated, all factors that can complicate attempts to follow a treatment regimen (4, 5). Because of its poor prognosis, the high cost of treatment, multiple physical symptoms, frequent hospitalizations, psychological duress, and social and occupational limitations, heart failure imposes great difficulties on the patients’ quality of life (4, 6–8). An impaired quality of life not only has adverse impacts on living but also enhances the risk of morbidity and mortality (9, 10).

Support in various aspects positively affects coping strategies and influences the well-being and quality of life of heart failure patients (11–13). This is closely associated with their behavior in coping with a new situation (6). Coping is a process in which an individual attempts to recreate a balance in his life through a series of stress-coping behaviors in response to a stressful incidence (14). The chronic nature of heart failure adds to the need for support (7, 8, 11). Support means informing and protecting an individual in order to help him or her make the best possible decisions, thus ensuring the observance of the patient's rights which must be perceived by him or her (15). In a recent review of the literature on heart failure, Koenig described social support as a coping resource that provides a clear path against stressors and problems associated with chronic diseases (16). Langford et al. described types of support, including 1) emotional support, which emphasizes love, empathy, and trust; 2) informational support, which gives the knowledge needed for problem solving; and 3) instrumental support which provides goods and services (17). Numerous studies have highlighted the relationship between received support and self-care in patients with heart failure. They indicated that patients who receive more support care more for themselves (7, 9, 18, 19). Murberg et al. suggested emotional support as an important mechanism for compliance to a treatment regimen and the prevention of depression symptoms (20). Several studies have found that the heart failure patient's perception of inadequate support may bring about reduced self-care abilities, poor compliance, depression, increased hospital readmissions, high-cost conditions, and adverse outcomes for the patient, his or her family, and even society (21, 22).

Support provides a new cultural context for health promotion (4). Understanding the cultural experiences and desires of patients, therefore, is essential to enabling one to offer appropriate nursing care. In many cases, hints can be found in the personal experiences of patients, the knowledge of which can help nurses offer services tailored toward obviating the patient's concerns by accurately identifying his or her needs and worries. Furthermore, the patient's understanding of the perceived support is intertwined with his or her culture, race, and ethnicity (23), and the perception of any support is different across different societies. In Iran, sources of support are limited, and patients suffering from chronic diseases are quite dependent on their families (24). No public associations exist to support heart failure patients, and Iranian patients do not receive home nursing care (25). Several studies have shown that the quality of life of Iranian heart failure patients is low with regard to the physical, psychological, social, and economic dimensions (26, 27). However, no studies have investigated the barriers to coping and types of support needs in heart failure patients. Therefore, the purpose of this study is to explore obstacles to coping related to support in heart failure patients as viewed by the patients themselves and their family members and caregivers.

## Methods

### Design

This qualitative study was conducted using content analysis. Qualitative research is an important tool for understanding emotions, perceptions, and information about the complexities of human responses which cannot be obtained through a quantitative study. Content analysis is a systematic coding-and-categorizing approach which involves a process of understanding, interpreting, and conceptualizing the underlying meanings of qualitative data (28).

### Sample and setting

Considering the qualitative nature of the data, the study environments were authentic. A total of 20 individuals (11 patients with heart failure, three cardiologists, three nurses, and three family members of heart failure patients) participated in this study. Patients and family members were selected from the physician offices and cardiovascular departments of Kerman teaching hospitals, the largest referral centers for cardiac patients in southeastern Iran. Interviews were conducted with heart failure patients and their family members in their homes after the researcher became acquainted with them, proposed the location, and got the consent of the patients. Interviews with cardiologists and nurses were conducted in the hospital at their convenience and with their consent after the researcher became familiar with each participant individually.

Participants were selected through purposive sampling, which was continued until data saturation was reached. Inclusion criteria for patients were a history of at least 6 months of symptomatic heart failure and no diagnosed psychological illness. Requisite criteria for family members were age of ≥18 years and an experience of having lived with a heart failure patient for at least 2 years. Nurses and cardiologists needed to have at least 2 years of experience caring for heart failure patients. All participants were required to maintain face-to-face communication in Persian with the researcher. Participants were selected such that maximum diversity was ensured. Interviews were performed individually, and each lasted 45–60 min.

### Data collection procedure

Open-ended, semi-structured interviews were employed for data collection. Initially, a few questions designed to acquaint the authors with the participants and create a friendly environment were asked. Then the interview was navigated toward the purpose of the study. Some of the posed questions were: ‘Since you developed the disease, what support problems have you experienced?’, ‘How did you cope with these problems?’, ‘Why is supporting patients with heart failure not satisfactory?’ Proportionate to the answers, in-depth and exploratory questions were put forth, like ‘Could you explain more?’ All interviews were conducted by the corresponding author and recorded with the participant's consent. Prior to commencing the interviews, the study purpose and method, confidentiality of data, and inclusion and exclusion rights were explained to the participants, who then signed a written informed consent form. Interviews were scheduled by participants such that their daily schedules were not disrupted. Data were collected up to the point of saturation, which was reached when no new data were observed regarding the phenomenon under study (29).

#### Data analysis

Data analysis was conducted according to the method proposed by Graneheim and Lundman (30). The aim of qualitative content analysis is to achieve a condensed and extensive description and understanding of the phenomenon (31). In the first step, every interview was transcribed verbatim and read through several times to obtain an overall understanding of the content. Second, the text was divided into meaning units that were condensed. Each meaning unit comprised words and sentences containing aspects related to each other. Third, the condensed meaning units were condensed and labeled with codes. In the fourth step, the codes were classified into subcategories and categories based on similarities and differences. A category consists of similar codes in the manifest level. Finally, the underlying meaning and content of the data were extracted, and themes were formulated as the expression of the latent meaning of a text. A sample of the process of analysis used in this study is shown in Table 1. During data collection and analysis, the researcher wrote down any reflections or hints related to the data in a memo to be used for future interviews.

**Table 1:** Example of qualitative content analysis process

Category	Subcategories	Open code	Meaning units
Inadequate family performance	Lack of emotional support	The psychological damage caused by irritable family members.	My daughter looks after me. She is impatient and gets angry easily. She treats me badly. That always breaks both my heart and my spirit. (Patient 3)
	Lack of physical care	Inability of wife as a barrier to patient care	My husband has a disability and can't help me in my daily activities. Actually, I am the one who must take care of him, and that makes it harder for me. (Patient 10)
	Lack of knowledge	Worsening of symptoms due to a lack of family consciousness	My daughter knew little about my disease. Though she was very kind, she didn't know how to take care of me. She gave me non-diet foods, wouldn't let me exercise; some time later I gained weight and the symptoms returned. My doctor was mad. (Patient 6)

The trustworthiness of the study was tested using Guba and Lincoln critera, expressed by Streubert and Carpenter (32). Accuracy and reliability of the data were ensured by checking the codes with the participants, revision by supervisors, and long-term involvement with the data, as the researcher was involved with the subject, data, and patients for over a year. The researcher visited each participant before the interview to build trust and to create the grounds for an in-depth interview. A portion of the text along with the initial coding was shown to the participant, who compared the degree of homogeneity between the ideas extracted by the researcher and his original opinions. Supervisor revision was obtained by presenting the concepts and classifications developed from the data to experts of qualitative research to control the degree of fitness until a consensus was reached. The corresponding author translated the categories and quotations from the interviews from Farsi into English accompanied by English native speakers, and the results were then fine-tuned by professional editors.

### Ethical considerations

This study was approved by the relevant ethical committee (number: k.93.246 /4.10.2014). Oral and written informed consent was obtained from participants before beginning the study and before the interviews were recorded. Participants were free to enter and exit the study at will and were assured of the confidentiality of the information. Participants were allowed by the researcher to call or e-mail regarding any questions or information.

## Results

Eventually, 11 patients with heart failure, three cardiologists, three nurses, and three family members of heart failure patients were interviewed. Six patients were men and five were women. Average participant age was 64 years, and most participants were married. Participants had 8 months–10 years of experience with heart failure at New York Heart Association functional classes III and IV. They had different educational backgrounds, ranging from illiterate to bachelor's degree, and most of them were retired. Cardiologists and nurses (three female cardiologists, two female nurses, and one male nurse) had an average age of 45 years and had 7–25 years of experience caring for heart failure patients. Family members (one wife, one daughter, and one daughter-in-law) had an average age of 38 years and 2–10 years of experience living with a heart failure patient.

After data analysis, the initial categories were given conceptual and abstract tags according to their nature. Thus, the natures and dimensions of the patient's perception of support obstacles were exposed, and the main theme ‘Defective support network: a major obstacle to coping for patients with heart failure’ was revealed. A defective support network refers to a set of factors that, from the perspective of the participants, hinder their ability to cope appropriately with their new situation. In fact, receiving support in various aspects, such as shelter, made patients adapt to the new situation and have a sense of well-being. Any defect in the network prevents patient compliance with the treatment regimen and reduces the quality of life. This theme included four subcategories: inadequate family performance, inadequate support of the healthcare team, distorted social support, and inadequate welfare support (Table 2). Each of the aforementioned categories is defined using direct quotes from the participants.

**Table 2:** Theme, categories, and subcategories

Theme	Categories	Subcategories
Defective support network	Inadequate family performance	Lack of emotional support Lack of physical care Lack of knowledge
	Inadequate support of the healthcare team	Lack of educational support Lack of emotional support Lack of physical support
	Distorted societal social support	
	Inadequate welfare support	

### Inadequate family performance

One recurring issue in this study was inadequate family performance, emotionally and physically. Lack of emotional support from family causes the greatest harm to patients. Because of the chronicity of heart failure, patients constantly face multiple symptoms and treatment problems. Clearly, they need a source of emotional support, such as a family. A lack of emotional support may also cause relapse of the adverse symptoms of the disease. A 75-year-old male patient said: ‘When I am left alone, I feel sick. I get shortness of breath, and everything goes wrong in my body. I expect my family to stay with me and listen to what I have to say, but when it's not like this, nothing else matters anymore’.

Since most heart failure patients are elderly and unable to take care of themselves, they need the support of family members for care and in performing daily activities. Inadequate family support in physical care could be a serious obstacle for patients. A 70-year-old female patient said: ‘Had I lived at my own house and my family looked after me, made me low-fat food, and made me take my medicine on time, I certainly would have been better than I am now’.

In some cases, poor family support resulting from a lack of proper knowledge interfered with the treatment process. Therefore, family support can be educational, and a lack of knowledge may give way to a series of problems for the patient. A cardiologist with 11 years of experience confirmed this by stating: ‘As a physician, when I am uncertain about the family, I can't prescribe many drugs for the adverse effects. The treatment will be limited, too. I just have to pick the drugs so that it doesn't put the patient at risk’.

The majority of patients emphasized family support as helping the heart failure patient achieve a better condition. The participating patients unanimously considered family support to be the most important need in the course of coping with the disease. A 61-year-old female patient said: ‘It is important to be loved. When I see that everyone loves me, I too begin to love my life’.

This need becomes clearer when, as acknowledged by some participants, patients with families in possession of correct information experience better conditions. To care for themselves, heart failure patients need to knowledge about self care. Knowledge increases trust and confidence in patients and prevents a relapse of symptoms. A 31-year-old female patient said: ‘My sister-in-law was a nurse. She gave me a reality check; she told me what is wrong with me and what I have to do to have a normal life. What she taught me has been very effective’.

A family member said: ‘My father-in-law was at peace knowing that I used to be a CCU nurse. I was his supporter and the one he relied on, and I always had my eyes on his symptoms and lifestyle’.

In this study, a distinctive role for the spouse in supporting the patient was observed; patients who either had no spouse or had a spouse who did not have the required knowledge were less satisfied. An 81-year-old male patient said: ‘My wife's death was the greatest stress on me; since the day I became alone, my problems have increased much more’.

The need for spousal support is highlighted when the patients regard it as the most important contributing factor to adjusting to the new situation. A 58-year-old male patient stated: ‘My wife was always next to me. She kept up with me and never broke my spirit. She encouraged me all the time. She gave me my medicine and looked after me’.

### Inadequate support of the healthcare team

One important need of patients with heart failure, reiterated regularly in their statements, was the need for the support of the healthcare team, that is, physicians and nurses, in a range of educational, emotional, physical, and reassuring aspects, defects in any of which would be a potential obstacle to patient coping. Since heart failure is chronic and knowledge of the disease and self-care have significant impacts on the course of treatment and the life of the patient, the most important defect in the performance of the healthcare team as viewed by the patients was not providing patients with proper instructions. A 63-year-old female patient said: ‘During the 3 years of my illness, nobody taught me anything. I acquired knowledge myself over the course of time. I learned by trial and error’.

Another worrying issue for patients was that the healthcare team did not provide the patient with sufficient information about the measures they provided, which was confusing for the patient. For example, a 63-year-old male patient said: ‘When the nurse is attending me without saying what she is doing, I get confused. I want to know what they are doing for me’.

The need for mental support from the healthcare team was yet another aspect greatly emphasized, the absence of which disrupted the course of treatment. A 43-year-old male patient confirmed this point: ‘Geniality and good manners in a nurse are very important to me. Sometimes you put all your hope in your doctor and nurse. When I see them frowning or grumpy, I get a bad feeling and my spirits sink’.

A nurse with 14 years of experience said: ‘Communication is effective in the patient's treatment and care; but, unfortunately, we are understaffed and don't have enough time to communicate’.

Another issue emphasized by the patients as affecting the course of their disease and treatment was their need to be reassured by the healthcare team, the lack of which was considered a huge obstacle to treatment. An 83-year-old male patient stated: ‘I have a fear of invisibility when being hospitalized. If I am hospitalized in a ward with no visibility and out of the line of sight of the nurses, I feel fear and insecurity’.

### Distorted societal social support

A further aspect of the support network, mentioned as a need, that is, support from society and non-related friends was said to play a significant role in the well-being of patients: A 75-year-old female patient said: ‘Even though I have heart failure, I still need to be seen in society. Society plays a great role in my coping with this situation of mine’.

Some factors, like age or even society's attitude, can further expose the vulnerability resulting from distorted *societal* social support. A 31-year-old female patient stated: ‘As a young woman, I used to hate going out, because the pathetic looks from people bothered me. I don't like to be looked at with pity; I want to be treated as a normal person when I go out’.

If social relationships are not maintained at a proper level or if they are extreme, they can have a reverse impact. One accepted tradition in some countries, including Iran, is visits to the patient by family and friends at the hospital or at home at the time of relapse, something which can cause a series of damages. A 63-year-old male patient said: ‘When I get too many visitors, I get a bad feeling, and I wonder if I'm dying’.

### Inadequate welfare support

An additional dimension of the support network, the defect of which severely afflicts the course of treatment and care in patients with heart failure, is economic support. Patients may suffer from economic problems in two ways. First, the disease underlies economic problems. Since heart failure is a debilitating disease, it seriously affects the patients’ economics, especially of those not yet retired and responsible for earning a living. Second, economic problems impede proper treatment and follow-up. A 43-year-old male patient said: ‘I was a construction worker. I was the breadwinner and not insured. I had problems getting my medicine; I had to keep on working. I worked a few days and was hospitalized again’.

Heart failure, due to its chronic nature, affects the way of life of patients and even their families, such that they endure many problems during their life with the disease which require the tangible support of health organizers, support organizations, and insurance companies. A 58-year-old male patient noted: ‘Unfortunately, my main problem was that I was not insured. My illness was difficult and costly. I also had to work for my children. I was a mere manual laborer, and my job was heavy work. I wasn't supported from anywhere’.

Given the importance of follow-up care and treatment in heart failure patients, it is necessary for authorities and organizers to establish a support association for such patients. In this case, a 31-year-old female patient stated: ‘Had there been an association, I would have enrolled. I managed to cope very well with my severe heart failure and a 20% heart, and have my normal life. I'd like other patients to hear my story and be hopeful and happy’.

## Discussion

Findings of the present study revealed that support, in all its aspects, is the most essential need of patients with heart failure, and any defect in the offered support is the greatest obstacle to coping appropriately with the new situation. Based on experiences of the participants, this defective support network was categorized into inadequate family performance, inadequate healthcare team support, distorted *societal* support, and inadequate welfare support. Findings from this study indicated that inadequate family performance in emotional and physical dimensions is a serious obstacle to coping for patients with heart failure. Familial emotional support is one of the basic needs of such patients and, if inadequate, will cause the greatest damage. This finding is consistent with the study by Maeda et al. (8) who indicated that effective family support leads to the self-efficiency of patients with heart failure. Patients perceiving their family's mental support benefit from greater self-confidence and not much prone to depression.

From the perspective of participants, lack of emotional support from family causes adverse effects like relapse and reduced quality of life. In their study, Årestedt et al. similarly concluded that the less the amount of perceived support by the heart failure patient is, the lower the patient's performance and the more severe the disease will be, and that supporting the patient leads to an increased quality of life, especially from an emotional perspective (13).

The present study showed that physical family support was a basic need of patients with heart failure. In confirmation of this, researchers have concluded that these patients need the support of their families in performing their daily activities, and hence, family members of these patients need to offer their effective support through emotional support, first-hand care, and facilitation of healthy behaviors (11, 12). Inadequate family support may, at times, make the physician doubt the patient's adherence to the drug regimen, and, consequently, the prescription of effective medicine. Several studies have similarly shown that sensitivity to medication adherence and survival is higher in married patients who benefit from family support (5, 7, 9).

This study determined that there is a need for spousal support, as it was much emphasized by the participants who maintained that patients bereft of spousal support sustain higher mental and psychological damage. This finding is consistent with numerous studies that concluded that mortality rates were higher in patients without spousal support, which was also associated with higher instance of depression and a reduced motivation for self-care (5, 7, 19). Luttik et al. showed that there is a considerable difference between the quality of life of married patients and those who live alone. In a 9-month follow-up, they showed that married patients experienced fewer complications and a minimal readmission rate (12). In different results obtained by Gallagher et al., the presence of a partner is not associated with a positive outcome in self-care behaviors. Patients who receive moderate levels of family support showed no difference between patients with and without partners (18). In the current study, when family support reached a certain level in multiple domains, it was deemed effective. Families who provide full and tangible support help the heart failure patients cope with their illness. In some cases, defective family support due to lack of adequate knowledge disrupted treatment and care for patients. Studies similar to this study concluded that poor family performance resulted in lower confidence level in patients, whereas strong and conscious family performance led to a higher level of confidence and adherence to the diets and drug regimens, which showed considerable progress in families trained in this regard (3, 4, 7).

This study viewed inadequate educational, emotional, and physical support, and lack of reassurance from the healthcare team for patients with heart failure as one of the greatest obstacles highlighted by the participants. The most important obstacle to support from the healthcare team was lack of effective and adequate instructions to patients with heart failure; such patients require knowledge of their disease and self-care principles to benefit from a high quality life. This finding was consistent with that proposed by Carr et al., who maintained that support is the requirement for an enhanced quality of life in patients with heart failure. Raising knowledge is, *per se*, an incentive for proper self-care; the absence of instruction causes poor self-care and frequent hospitalizations and, as a result, creates an obstacle to coping with the new situation (21).

Since patients with heart failure live with their disease for a long time, it is imperative that they are provided with information on their course of treatment, so that they are reassured about the conducted actions and also enabled to make appropriate decisions. One major worry of the participants of the present study was the confusion raised by ignorance about medical and nursing measures. In confirmation of the said finding, Albert et al. found that patients with heart failure need to have accurate information about their situation, and the caring nurse also needs to have adequate relevant knowledge, which must be conveyed to the patient in a calm manner, with proper motivation and allocated time (22).

Based on the current results, perceived poor emotional support from the healthcare team is a serious obstacle to coping for patients with heart failure. In a similar study, Riegel and Carlson concluded that defective mental and psychological support of the caring team reduces the quality of life and causes frequent hospitalizations (19).

Along with inadequate support of the healthcare team, participants also emphasized the building of trust by the healthcare team, as, considering the nature of the disease, patients are in need of regular referral and follow-up and are, sometimes, hospitalized due to relapse. Therefore, trusted relationships with the healthcare team, especially nurses, is a significant help to the patients. Furthermore, results showed that a lack of trust in the healthcare team is a major obstacle in the path of treatment and care for these patients. As Jurgens et al. claimed, this uncertainty causes stress in patients and delays in referral and treatment follow-up (33). In this study, it was found that higher certainty and trust were reasons why patients were more satisfied with admission to the coronary care unit (CCU). There, nurses have greater experience and skills and more control over the patients, made possible through the building structure, compared with the emergency room or other wards. Thus, patients with heart failure need support from the healthcare team for the use of problem-focused strategies to cope with their new situation.

The next major obstacle to coping is defective *societal* support, defined here as the support an individual perceives from the society in which he or she lives (excluding family members and the healthcare team). As indicated by the results, patients with heart failure, especially the younger ones, due to the newly found chronic disability become restricted in their activities. Gradually, they withdraw from society, friends, and family; this isolation will have a destructive psychological effect on the patient, and even if family support is present, he or she will feel depressed and sick. In support of these results, Eng et al. stated that patients who experience social isolation are exposed to morbidity and mortality risks two to four times more than those with more relationships with friends and the society in which they live (34). Social relationships of patients with friends and favorite people creates in them peace and motivation. In recent studies, it was revealed that the social relationships of patients with heart failure motivated them and increased their quality of life (12, 13). Findings showed that inappropriate social reactions, such as pathetic and demeaning looks, make the individual avoid social relations. Nasib et al. maintain that, since humans are social beings, social relations serve to protect and develop their dignity. Society needs to be devoid of demeaning or pathetic attitudes toward minorities, like special patients, and thereby encourage their presence in society. Looks, tags, and differences must not disrupt the active presence of these patients in society (35).

The custom of maintaining ties with families in some societies like Iran encourages frequent visits at the time of relapse, when the patient is not in a particularly comfortable condition and needs rest and treatment. Such visits merely annoy the patient rather than calm him or her. Patients preferred to have such relations when they were in a stable condition. There are different results for this. Mahdavi Shahri et al., for instance, showed that, as stated by CCU nurses, visits are beneficial to the patient (36). On the contrary, nurses in the study by Berti et al. regarded visits as barriers to feelings of comfort in patients (37). Of course, neither of the above studies assessed patient experience, and the provided opinions can always be agents of the governing cultures. Additional studies on the understanding and experiences of patients are required to further clarify this concern.

Yet another result of this study was the effect of inadequate welfare support as an obstacle to coping of patients with heart failure, especially in younger patients and in those responsible for earning their living. Numerous studies have confirmed this fact and concluded that low economic status in patients with heart failure negatively affects their quality of life, and frequency of hospitalizations, undesirable feelings, self-care, and mortality rates are considerably higher in these patients (10, 23).

Most of the patients with heart failure were concerned about the lack of coordination of services in the health and treatment system. Patients in small towns and villages received even less care and had to undergo hardships and further expenses to receive treatment in larger health centers. Joynt et al. concluded that less-equipped hospitals with poor management and planning fail to offer desirable care. As a result, readmissions and adverse effects occur more frequently in patients referred to these centers (6).

The absence of support associations was another obstacle affirmed by participants who maintained that the existence and support of an association would enable heart failure patients to exchange experiences and instructions and, consequently, facilitate their coping with the disease. In support of this, Riegel et al. asserted that learning from peers can effectively contribute to the outcomes of living with the chronic disease of heart failure (4).

This study encountered a limitation. Despite the efforts of the researchers to select a most diverse group of participants, the study lacks the required racial and cultural diversity. Therefore, the provided answers might not represent the general population of patients with heart failure.

## Conclusion

The findings of this study extend the understanding of obstacles to coping of patients with heart failure. Across all stages of heart failure, from the onset of affliction through to the next stages, patients need to perceive support from different quarters. Not all of these needs can be met by the family. Indeed, the society, the healthcare team, and the authorities backed by commitment and efficient support regulations are required to assist patients proportional to their support needs. In doing so, they are enabled to cope with their new situation effectively and experience a high quality of life. They must also contribute to the prioritization of care in patients with heart failure and how they are treated and interacted with in a range of different dimensions.

The results of this study suggest that knowing obstacles to coping of patients with heart failure can help healthcare providers plan educational and supportive interventions for promoting coping strategies and quality of life in these patients. Since coping strategies and providing support are influenced to a large extent by culture, it is recommended that further studies in this regard be done in other communities.
